# Smartphone-Based Pupillometry Using Machine Learning for the Diagnosis of Sports-Related Concussion

**DOI:** 10.3390/diagnostics14232723

**Published:** 2024-12-03

**Authors:** Anthony J. Maxin, Bridget M. Whelan, Michael R. Levitt, Lynn B. McGrath, Kimberly G. Harmon

**Affiliations:** 1 Department of Neurological Surgery, University of Washington, Seattle, WA 98195, USA; ajm39354@creighton.edu (A.J.M.); mlevitt@uw.edu (M.R.L.); 2 School of Medicine, Creighton University, Omaha, NE 68178, USA; 3 Department of Family Medicine, Sports Medicine Section, University of Washington, Seattle, WA 98195, USA; 4 Departments of Radiology, Mechanical Engineering, Stroke & Applied Neuroscience Center, University of Washington, Seattle, WA 98195, USA; 5 Department of Neurological Surgery, Northern Light Health, Portland, ME 04102, USA

**Keywords:** smartphone pupillometry, sports-related concussion, diagnostics, biomarkers, pupillary light reflex, digital health

## Abstract

**Background:** Quantitative pupillometry has been proposed as an objective means to diagnose acute sports-related concussion (SRC). **Objective:** To assess the diagnostic accuracy of a smartphone-based quantitative pupillometer in the acute diagnosis of SRC. **Methods:** Division I college football players had baseline pupillometry including pupillary light reflex (PLR) parameters of maximum resting diameter, minimum diameter after light stimulus, percent change in pupil diameter, latency of pupil constriction onset, mean constriction velocity, maximum constriction velocity, and mean dilation velocity using a smartphone-based app. When an SRC occurred, athletes had the smartphone pupillometry repeated as part of their concussion testing. All combinations of the seven PLR parameters were tested in machine learning binary classification models to determine the optimal combination for differentiating between non-concussed and concussed athletes. **Results**: 93 football athletes underwent baseline pupillometry testing. Among these athletes, 11 suffered future SRC and had pupillometry recordings repeated at the time of diagnosis. In the machine learning pupillometry analysis that used the synthetic minority oversampling technique to account for the significant class imbalance in our dataset, the best-performing model was a random forest algorithm with the combination of latency, maximum diameter, minimum diameter, mean constriction velocity, and maximum constriction velocity PLR parameters as feature inputs. This model produced 91% overall accuracy, 98% sensitivity, 84.2% specificity, area under the curve (AUC) of 0.91, and an F1 score of 91.6% in differentiating between baseline and SRC recordings. In the machine learning analysis prior to oversampling of our imbalanced dataset, the best-performing model was k-nearest neighbors using latency, maximum diameter, maximum constriction velocity, and mean dilation velocity to produce 82% accuracy, 40% sensitivity, 87% specificity, AUC of 0.64, and F1 score of 24%. **Conclusions**: Smartphone pupillometry in combination with machine learning may provide fast and objective SRC diagnosis in football athletes.

## 1. Introduction

Sports-related concussions (SRCs) are common, with an estimated 1.0–1.8 million per year in the 0–18 years age range [1] and 6.2% of college football players sustaining a SRC annually [2]. The diagnosis of SRC can be difficult [3]. The most sensitive and specific measure for diagnosis of concussion is symptoms; however, this requires the athlete to report symptoms [4]. An athlete may not report symptoms because of internal or external pressures, unrecognized symptoms, or a delay in the development of symptoms [5]. The Sport Concussion Assessment Tool (SCAT) is recommended for the diagnosis of concussion and contains both subjective and objective portions. The subjective portion includes a symptom scale where users rate each of 22 symptoms associated with concussion on a Likert scale from 0 to 6. The Symptom Score represents the number of symptoms present (from 0–22) and a Symptom Severity Score is the sum of the symptoms endorsed on the Likert scale (0–132). Symptom score and symptom severity score are the most predictive of concussion with an AUC of 0.93–0.94; however, symptoms are subjective and rely on accurate reporting by the athlete [4]. An athlete may under-report symptoms due to a desire to return to play, concern about letting down teammates, pressure from coaches, an inability to recognize symptoms, or a delay in the development of symptoms. The SCAT-6 also has a cognitive evaluation, (the Standardized Assessment of Concussion (SAC)), and a balance evaluation (the modified Balance Error Scoring System (m-BESS)) [6], but the sensitivity and specificity of both these tests are poor [4,7,8]. Other tests which have been used include the King–Devick and computer-vision eye-tracking devices [9,10] and blood biomarkers but all have challenges related to their accuracy, practicality, and usability [11]. Currently the diagnosis of concussion relies heavily on self-report of symptoms. There is a need for a quick, accurate, easy-to-use objective biomarker of concussion.

Pupillometry has been explored as one such biomarker. Our pupils change in size continuously in response to the ambient light levels in a complex reflex known as the pupillary light reflex (PLR). The PLR is modulated by both sympathetic and parasympathetic input and not under conscious control. The PLR is affected by age, sex, attentional state, ambient light, and other factors [12]. The PLR was first reported as an indicator of health in the ninth century and qualitative pupillometry is a core component of the Glascow Coma Scale [12]. Quantitative pupillometry has more recently been used in intensive care units and emergency rooms as a more accurate assessment of pupil metrics. Studies of the PLR in the military in those with blast injury or chronic mild traumatic brain injury (mTBI) showed decreases in PLR and pupil size compared to uninjured controls 15 days to greater than a year after injury [13,14,15] and more acutely at <72 h after injury [16]. In a study of 92 youths with a diagnosis of post-concussion syndrome, the velocity of the PLR was increased compared to uninjured controls at a median of 51 days post injury [17]. Another study of adolescents aged 12–18 at a median of 12 days after SRC showed group level increases in PLR metrics [18]. These studies all used a NeurOptics pupillometer, a quantitative pupillometer often used in intensive care units [19]. The direction and magnitude of PLR changes in these studies was not consistent; however, interest in quantitative pupillometry as an objective biomarker of concussion has been piqued by these initial studies. Additionally, although the pathophysiologic mechanism behind pupil changes in the setting of SRC and mTBI is unknown, it is thought to result from functional rather than structural abnormalities in neuronal homeostasis that are the basis of mTBI pathophysiology [20].

There has also been interest in utilizing the capabilities of smartphones to quantitively measure the PLR [20,21,22]. Smartphones are ubiquitous and easy to use. A smartphone-based app with the ability to differentiate concussed from non-concussed athletes objectively would present a significant advancement in the diagnosis of concussion. A recent pilot study in mTBI patients presenting to the emergency room with either loss of consciousness or memory loss showed that a machine learning algorithm combined with smartphone pupillometry was able to differentiate between those with mTBI and healthy controls [23]. SRCs are a subset of mTBI on the milder end of the spectrum [3] with loss of consciousness or memory loss occurring in only 5% and 10%, respectively, in college athletes [24]. We studied the utility of a smartphone pupillometry application with machine learning (PupilScreen, Apertur Inc., Seattle, WA, USA) for the detection of acute SRC in college football athletes.

## 2. Methods 

### 2.1. Data Collection 

Participants included Division I collegiate football players from a single institution between 6 August 2023 and 8 January 2024. Every player on the football team was eligible to participate in the study. All participants underwent screening for baseline anisocoria by the senior author prior to baseline smartphone-based quantitative pupillometry testing during pre-season training camp. Individuals diagnosed with SRC underwent the same pupillometry testing within 24 h of when the SRC occurred. The diagnosis of SRC was made by a team physician using the definition from the Amsterdam International Consensus Conference [25]. Each athlete completed the Sport Concussion Assessment Tool 6 (SCAT-6) both at the time of their baseline pupillometry recording and their concussion pupillometry recording. Both symptom score and symptom severity score at baseline and post-concussion were reported for concussion and controls. Eye color and any concussion co-morbidities were also recorded at the time of enrollment. All athletes completed electronic informed consent. This study was approved by the University of Washington Human Subjects Division. 

**Figure 1:**
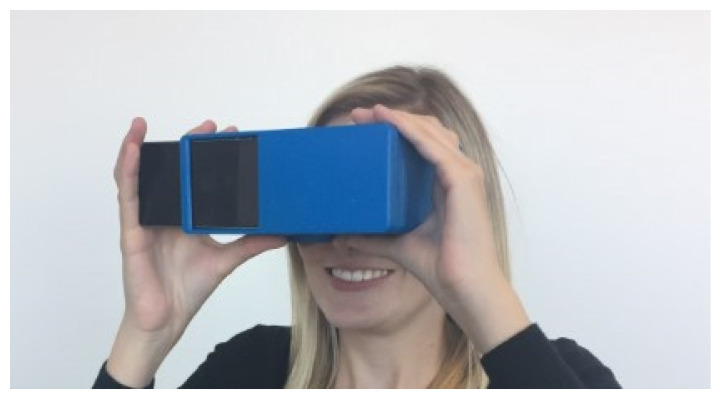
Demonstration of use of the box apparatus. The smartphone inserts into the box from the side (Mariakakis et al. [30]).

The PupilScreen smartphone-based quantitative pupillometry application (Figure 1) records a PLR curve along with seven PLR parameters representing the curve morphology (Table 1) using a computer vision algorithm trained on thousands of pupils to detect the pupil diameter throughout the recording. Each recording is binocular and eight seconds in duration, with a three-second flash of light from the smartphone camera in the middle of the recording to stimulate the PLR [26,27,28,29]. During this study, a 3D-printed box apparatus was attached to the smartphone for each recording to eliminate the effect of ambient light on the pupillometry results, and there is no illumination source in the 3D-printed box prior to the light stimulus (the baseline maximum pupil diameter before the light stimulus is recorded in the latency period between the onset of light stimulus and the beginning of the pupil constriction—see Limitations Section for further discussion) [23]. The PupilScreen emits light intensity equivalent to 1.1 candela at the plane of the cornea. The iPhone version 12 was used for all recordings.

### 2.2. Analysis 

Football position played, presence of comorbidities, and eye color were analyzed using a Fisher’s exact test. Age, year in school, and baseline concussion symptom reporting were analyzed using a Mann–Whitney U/Wilcoxon Rank Sum Test. History of concussion was analyzed using a logistic regression. For all statistical analyses, a *p*-value of 0.05 was considered as the threshold to determine statistical significance.

Descriptive demographic and pupillometric data were produced, including effect sizes for each of the PLR parameters in isolation and single-variable area under the curve (AUC) calculations for each PLR parameter in isolation. All combinations of the seven PLR parameters (Table 1) were tested in machine learning binary classification models to determine the optimal combination for differentiating between athlete baseline recordings and athlete recordings taken immediately after concussion. To conduct this method of analysis, all possible combinations of the seven PLR parameters (Table 1) were generated in a non-repeating fashion (i.e., the combination ‘latency, maximum diameter, mean constriction velocity’ is not repeated if the combination ‘maximum diameter, mean constriction velocity, latency’ is already present). These unique combinations were then tested sequentially as feature inputs in the machine learning classification model architectures that are subsequently listed in this text. 

Four machine learning model architectures were tested: logistic regression, k-nearest neighbors, support vector machine, and random forest [30]. Each was tested with and without the synthetic minority oversampling technique (SMOTE) which was employed prior to training of the machine learning models due to significant class imbalance in the dataset which can otherwise make the results of machine learning classification performance unreliable and impractical [31]. This technique oversamples the minority class (in this case, recordings from athletes immediately after concussion) within its statistical distribution to produce a new sample that is equal in size to the majority class. In the present study, this simulates the effect of collecting recordings on concussed athletes for approximately nine seasons at the rate collected within this study of 11 concussed athletes per football season. SMOTE thus generates a dataset on which the machine learning architectures can be tested to see what model performance would look like if a balanced dataset was collected (i.e., a dataset with equal numbers of concussed and non-concussed athletes) which is otherwise not feasible to collect due to time constraints (it would take nine seasons to collect enough concussion data on our study population, see above). 

Ten-fold cross-validation stratified by cohort was used to produce the following model performance metrics when SMOTE was used: overall accuracy, sensitivity, specificity, area under the curve (AUC), and F1 score. The 10-fold cross-validation technique splits the dataset into 10 equal subsets and trains the model on nine out of 10 subsets with the 10th subset held out as a testing set. The performance is then recorded, that model is discarded, and the process is repeated nine more times and the model performance metrics are averaged across all 10 runs for each possible unique combination of the seven PLR parameters (Table 1) for each of the four machine learning model architectures to produce the performance metrics that are reported in this text. By averaging the performance over 10 folds, this approach gives an unbiased and accurate report of the expected model performance on an unseen testing dataset when one is not yet available, as is the case in this study. Five-fold cross-validation stratified by cohort was used to report the same model performance metrics in the non-SMOTE dataset due to the large class imbalance that was present. Due to the class imbalance, the model fitting and results of the non-oversampled dataset may be unreliable [31]. We report the best-performing feature combinations (i.e., combinations of PLR parameters) for the top two models, based on AUC value, in differentiating PLR curves of athletes with concussion versus baseline recordings for both the non-SMOTE and SMOTE model training and testing runs.

## 3. Results 

There were 93 football athletes (100% male) that had baseline pupillometry recordings taken with the smartphone pupillometry application. Eighteen athletes had a documented prior diagnosis of at least one concussion an average of 640 (QR: 306, 1029) days prior to receiving a baseline pupillometry recording. Ten percent of athletes had a diagnosed mood disorder (including depression and anxiety), 2% had a diagnosis of ADHD, and 1% had a diagnosed migraine disorder. There was representation across all football positions on the team. Eye color was not significantly different between concussed and baseline cohorts. Demographic and position characteristics are shown in Table 2. At the time of baseline pupillometry recording, the median SCAT-6 symptom score was 2 (IQR: 6) and the median SCAT-6 symptom severity score was 2 (IQR: 7). Eleven athletes sustained subsequent concussions during the study period and received additional concussion testing and pupillometry recordings immediately after injury. After the initial injury, the median SCAT-6 number of symptoms reported was 15 (IQR: 11.5) and the median SCAT-6 symptom severity reported was 25 (IQR: 30). 

Descriptive pupillometry data from our cohort prior to oversampling are presented in Table 3 with comparison to the oversampled data. The MIN PLR parameter had a moderate effect size when used alone to differentiate between baseline and concussion recordings. Without oversampling, the MIN, LAT, and MDV PLR parameters each in isolation had positive predictive ability better than chance based on their AUC values.

**Figure 2:**
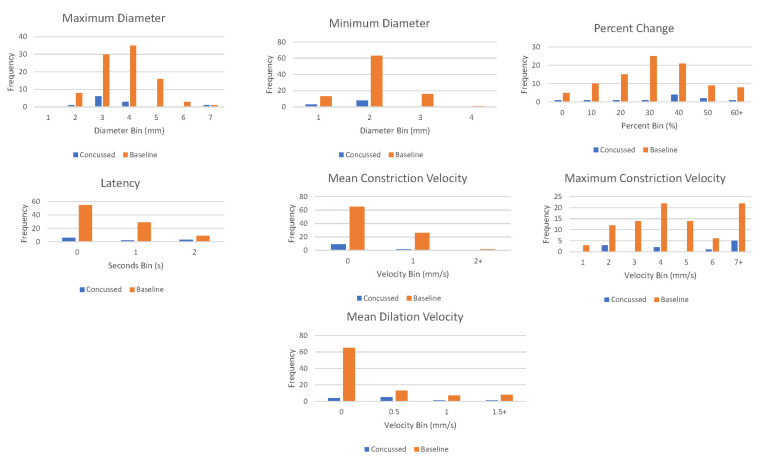
Double histograms of raw data for each pupillary light reflex parameter.

**Figure 3:**
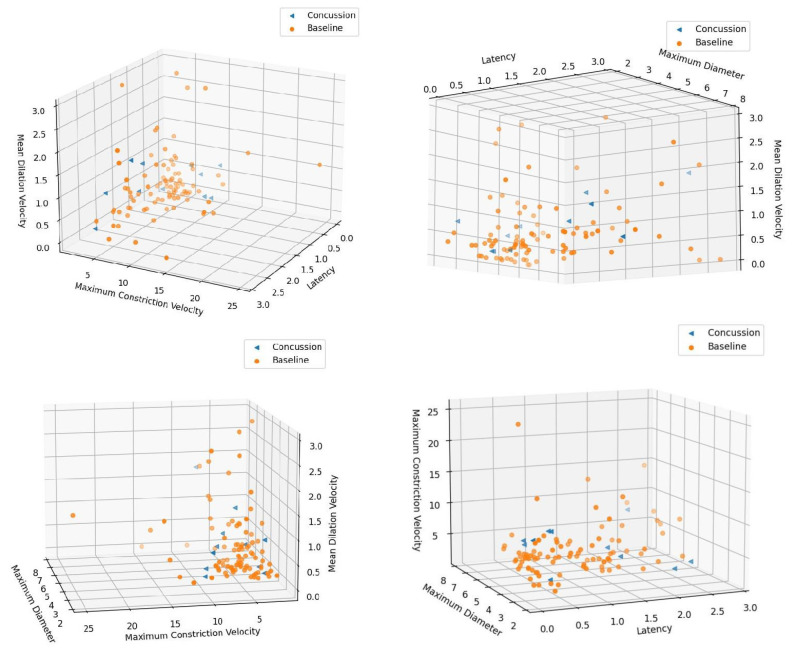
Three-D scatter plots comparing raw data from different combinations of three out of the four PLR parameters in the best-performing model without SMOTE. Views have been adjusted to give the best appearance of potential areas of differentiation between concussed and baseline recordings in our dataset.

For the machine learning pupillometry analysis, the single best-performing models for each model architecture for both the non-SMOTE and the SMOTE training and testing runs are listed in Table 4 and Table 5, respectively. Overall, the best-performing model was a random forest algorithm after SMOTE with the combination of LAT, MAX, MIN, MCV, and MAXCV PLR parameters as feature inputs. This model produced 91% overall accuracy, 97% sensitivity, 86% specificity, area under the curve of 0.91, and an F1 score of 92% in differentiating between baseline and SRC recordings in the balanced dataset. In the non-SMOTE highly imbalanced dataset, the best-performing model was a k-nearest neighbors approach with the combination of LAT, MAX, MAXCV, and MDV PLR parameters as feature inputs. This model produced 82% overall accuracy, 40% sensitivity, 87% specificity, an AUC of 0.64, and an F1 score of 24%. With the use of five-fold cross-validation, in the non-SMOTE dataset each fold has only one or two concussion recordings in the test set which explains the poor average sensitivity and F1 score (e.g., if the model incorrectly classifies the one concussion recording that is present in a given fold, that fold ends up with 0% sensitivity). Double histograms in Figure 2 and three-dimensional scatter plots in Figure 3 help visualize the potential areas of separation between the concussed and baseline recordings in our non-SMOTE dataset using combinations of three out of the four PLR parameters from the aforementioned k-nearest neighbors model.

## 4. Discussion 

The diagnosis of concussion can be challenging with currently recommended objective tests lacking in sensitivity and specificity [4,7,8]. Although a report of increased symptoms after a potentially concussive event is highly accurate for the diagnosis of concussion, athletes may be reluctant to report symptoms or symptoms may develop after the initial injury [4,8,32,33]. Objective tests do not require self-reporting. Currently recommended objective tests include a cognitive test, the SAC, and a balance test, the m-BESS [34]. The sensitivity and specificity of the SAC using a 10-word list for immediate memory and delayed recall was recently reported as 40% and 86% for a 4-point decline in overall score with an AUC of 0.70 [4]. Likewise, the m-BESS had a sensitivity of 40% and specificity of 61% for an increase of three balance errors and an AUC of 0.71 [8]. Other objective tests have been studied for their accuracy in the diagnosis of concussion including the King–Devick, a rapid number-naming test which had a sensitivity and specificity of 85% and 76% with AUC of 0.78 in one study [8], although it has not performed as well in other populations [35,36]. Computer vision eye movement tracking has also been proposed as an objective screening test although it did not differentiate between concussed and controls in one study [8]. An accurate, easy-to-use, objective test that can be used on the sideline would be a significant advance. The smartphone-based quantitative pupillometry application used in this study, which employs a computer vision algorithm and machine learning, had a pre-SMOTE accuracy of 82%, sensitivity of 40%, specificity of 87%, AUC of 0.64, and F1 score of 24% and a post-SMOTE accuracy of 91%, with 98% sensitivity, 84% specificity, and an AUC of 0.91 and F1 score of 92% when comparing the PLRs of athletes after SRC compared to baseline recordings of all athletes. 

Previous studies using a medical grade pupillometer have shown differences in the PLR metrics. A study of 20 warfighters with mTBI due to blast injury demonstrated decreases in LAT, ACV, ADV, and 75% recovery time (T75) compared to uninjured controls at 15–45 days post-injury [13]. Likewise, a study of 17 non-blast-injured individuals with chronic mTBI at least one year post-injury showed decreased ACV and ADV compared to 15 uninjured visually normal controls [14]. Pupillometry also showed decreases in ACV, ADV, and T75 more acutely (<72 h) in 100 soldiers with acute blast injury compared to 100 controls [16]. Conversely, 98 adolescents 12–18 years old with concussion showed increases in AVC, AVD, PDV, and T75 compared to 138 controls a median of 12 days post-injury [18]. More recently, a study in adolescents 5–11 and 12–18 years old found limited significant associations in pupillary metrics between those with pediatric concussion and controls [37] and a study comparing pupillary metrics in adolescents with sports-related concussion in the past 28 days to controls found limited differences in PLR metrics [38]. Thus, this significant variability in normal pupillary dynamics and PLR changes with age may confound traditional analytic approaches. The advantage of machine learning approaches is that multiple metrics can be considered simultaneously and in concert with one another as feature inputs for the model to produce a disease-specific classification using the PLR. 

A machine learning approach allows for the complex and dynamic relationships that exist within the PLR to be leveraged for disease classification in a way that would not otherwise be possible with the human eye or traditional analytic techniques alone. In this study, we investigated four unique machine learning model architectures (logistic regression, support vector machine, k-nearest neighbors, and random forest) [27]. The random forest model architecture was the most effective for distinguishing between baseline recordings and concussion recordings in our post-SMOTE cohort, while a k-nearest neighbors approach was most effective in our pre-SMOTE dataset and the proximity of concussed PLR recording ’neighbors‘ can be visualized in three dimensions in Figure 2 (although the actual model used four dimensions) [27]. The approach of combining multiple variables (in this case, PLR parameters (Table 1)) to detect differences between cohorts is novel in the concussion and pupillometry literature. This approach allows for more powerful classification and discrimination between difficult-to-differentiate cohorts (such as athletes with and without concussion) with an aim towards individual subject classification and diagnostic capability in the future that is not otherwise possible when comparing individual PLR parameters in isolation. One potential consequence of this approach is a relative lack of interpretability in how exactly the machine learning model uses the PLR parameter feature input variables to arrive at a disease-specific diagnosis (concussed versus baseline, for example). We believe that with the advent of generative artificial intelligence and mainstream applications of machine learning models in society, adverse reactions to placing increased trust in models such as ours that is applied in this study will decrease in the future.

Initial research on mTBI presenting to the emergency room using the PupilScreen app and machine learning showed the ability to discriminate concussed from controls with an overall accuracy of 93.5%, sensitivity of 96.2%, specificity of 90.9%, area under the curve of 0.936, and F1 score of 93.7% [23]. In that study, there were only 12 concussed participants and SMOTE was again used and the population was older (54.1 years) and had more severe injury than this cohort with all but one having a loss of consciousness and all but one having memory alterations [23]. However, in the current study we observed similar discriminatory ability using the PupilScreen app, despite less severe injury (SRC) compared to mTBI. 

Current medical-grade quantitative pupillometry devices are expensive [39] and may not be affordable or readily accessible to colleges, high schools, or youth sports, limiting their use. In contrast, smartphone-based quantitative pupillometry improves accessibility in these contexts, as well as in the underserved or remote populations that are most in need of an objective biomarker of neurological status for the wide variety of disease [23,24,40] and functional states [41,42,43] in which pupillometry has been studied. The ability to add machine learning-based diagnostics to the smartphone is another benefit because it could bring increased ability for objective diagnosis of SRC to the hands of trainers, physicians, and coaches. Despite variability in smartphone models, the light stimulus intensity emitted by a smartphone pupillometer can be controlled via the smartphone software and thus standardized across smartphone models and cameras.

This study does have limitations. There were only 11 athletes with SRC and 93 athlete baseline recordings; thus, we found it necessary to alleviate this class imbalance by using the SMOTE [28] algorithm. As this algorithm oversamples within the distribution of the reference minority class sample, this inherently presumes that the 11 athletes with SRC represent the range of PLR in football athletes with SRC. This assumption may be proven incorrect in future studies with larger cohorts; however, the use of SMOTE is the best option for this dataset and perhaps can help to justify larger studies in the future. We have also thoroughly presented the pre-SMOTE results of our imbalanced dataset of 93 baseline pupillometry recordings and 11 concussed pupillometry recordings to allow for complete transparency in our results. Another potential limitation of this study is the use of the box attachment [26] to the smartphone during the pupillometry recordings. This helps to standardize the distance of the phone from the pupils; however, the dark environment within the box when the light stimulus is not present from the phone camera flash makes it difficult to detect the MDV PLR parameter (i.e., the re-dilation of the pupil once the light stimulus is turned off). Future studies without the use of the box attachment could allow for better calculation of this parameter by the computer vision pupil-detection models deployed by PupilScreen which could lead to improved classification model performance.

Finally, this pilot study was only conducted on Division I male football players. Football was selected because it has a both high incidence of concussion and a large number of athletes. This study required immediate (most within 2 h, all within 24 h of injury) assessment and medical personnel and equipment were available in this higher resourced sport. There were no females on the football team. Results may be different in other populations such as youth, high school, or professional athletes or female athletes. Future work will include testing this model on another dataset and assessing performance in both football players and other populations including other athletes of diverse genders and sports such as soccer and women’s volleyball.

## 5. Conclusions

Accurate, objective testing for acute SRC is needed. Currently recommended testing modalities rely on self-report of symptoms or their accuracy is poor. Smartphone pupillometry in combination with machine learning provided fast and objective concussion testing in a small study of football athletes and was able to differentiate concussed from control athletes. Additional studies in larger and more diverse cohorts should be conducted.

## Figures and Tables

**Table 1:** Definitions of pupillary light reflex parameters.

Pupillary Light Reflex Parameter	Description
Latency (s) (LAT)	Time from onset of light stimulus to initial pupillary constriction
Percent Change (%) (CHANGE)	Percent change in pupillary diameter from maximum to minimum
Minimum Pupillary Diameter (mm) (MIN)	Minimum diameter after light stimulus
Maximum Pupillary Diameter (mm) (MAX)	Average resting diameter prior to light stimulus
Mean Constriction Velocity (mm/s) (MCV)	The average speed at which the pupil constricts after the light stimulus until the minimum diameter is reached
Maximum Constriction Velocity (mm/s) (MAXCV)	The maximum speed at which the pupil constricts after the light stimulus until the minimum diameter is reached
Mean Dilation Velocity (mm/s) (MDV)	The average speed at which the pupil dilates after removal of the light stimulus

mm: millimeters, s: seconds.

**Table 2:** Demographics.

	Total *n* (%)	Concussed *n* (%)	Baseline *n* (%)	Difference Between Groups (*p*-Value)
	*n* = 93	*n* = 11	*n* = 93	
Age (mean, range) ^+^	20 (18–24)	21 (18–23)	20 (17–24)	0.60 ^c^
**Position**				0.63 ^b^
Defensive backs	16 (17%)	2 (18%)	14 (17%)	1 ^b^
Linebackers	16 (17%)	4 (36%)	12 (15%)	0.09 ^b^
Defensive Lineman	8 (9%)	0 (0%)	8 (9%)	0.59 ^b^
Offensive Lineman	19 (20%)	3 (27%)	16 (20%)	0.69 ^b^
Skill positions (running back, wide receiver, tight end)	22 (24%)	2 (18%)	20 (24%)	1 ^b^
Quarterbacks	5 (5%)	0 (0%)	5 (6%)	1 ^b^
Specialists (kicker, punter, long snapper)	7 (8%)	0 (0%)	7 (9%)	0.59 ^b^
**Comorbidities**				0.08 ^b^
Mood	9 (10%)	3 (27%)	6 (7%)	0.01 * ^b^
ADHD	2 (2%)	0 (0)	2 (2%)	0.99 ^b^
Headache/migraine	1 (1%)	0 (0)	1 (1%)	0.99 ^b^
**Year in School**				0.75 ^c^
1	36 (39%)	4 (36%)	32 (39%)	1 ^c^
2	32 (24%)	3 (27%)	29 (35%)	0.91 ^c^
3	21 (23%)	3 (27%)	18 (22%)	0.84 ^c^
4	8 (9%)	1 (1%)	7 (9%)	1 ^c^
**Eye Color**				0.28 ^b^
Blue	16 (17%)	0 (0)	16 (20%)	0.20 ^b^
Brown	61 (66%)	10 (91%)	51 (62%)	0.09 ^b^
Green	10 (11%)	1 (9%)	9 (11%)	0.99 ^b^
Hazel	6 (6%)	0 (0)	6 (7%)	1 ^b^
**History of Previous Concussion ^‡^**				0.13 ^d^
1	16 (16%)	4 (36%)	12 (15%)	0.99 ^d^
2	2 (2%)	2 (18%)	0 (0)	0.99 ^d^
**Baseline Symptom Reporting**				
Total Symptoms (median, range)	2 (0–21)	2 (0–19)	2 (0–21)	0.64 ^c^
Symptom Severity (median, range)	3 (0–65)	2 (0–24)	3 (0–65)	0.71 ^c^

* Significant. ^+^ Age at time of test. ^‡^ Not including concussions sustained during this study period. ^b^ Fisher’s Exact Test. ^c^ Mann–Whitney U/Wilcoxon Rank Sum Test. ^d^ Logistic Regression.

**Table 3:** Descriptive pupillometry data.

PLR Parameter	Baseline Mean ± SD (*n* = 93)	Concussed Mean ± SD (*n* = 11)	Effect Size for Baseline to Concussion	Single-Variable AUC for Baseline to Concussion Before SMOTE	Single-Variable AUC for Baseline to Concussion After SMOTE
MAX	4.3 ± 1.1	4.1 ± 1.3	0.2	0.5	0.59
MIN	2.6 ± 0.5	2.3 ± 0.5	0.6	0.53	0.67
CHANGE	36.4 ± 15.9	38.4 ± 20.9	0.1	0.44	0.64
LAT	1 ± 0.8	1.1 ± 1	0.1	0.54	0.62
MCV	1 ± 1.1	0.8 ± 0.2	0.3	0.43	0.6
MAXCV	5.6 ± 3.5	6.2 ± 2.8	0.2	0.44	0.67
MDV	0.5 ± 0.7	0.6 ± 0.5	0.2	0.61	0.75

PLR: pupillary light reflex, SD: standard deviation, AUC: area under the curve using a random forest model, SMOTE: synthetic minority oversampling technique, MAX: maximum diameter, MIN: minimum diameter, CHANGE: percent change, LAT: latency, MCV: mean constriction velocity, MAXCV: maximum constriction velocity, MDV: mean dilation velocity.

**Table 4:** Best-performing PLR parameter combinations and models in the unbalanced dataset before SMOTE.

Model	Parameters	Accuracy	Sensitivity	Specificity	AUC	F1 Score
KNN	LAT, MAX, MAXCV, MDV	82%	40%	87%	0.64	24%
RF	MDV	86%	30%	93%	0.61	28%
SVM *	-	-	-	-	-	-
LR *	-	-	-	-	-	-

RF: Random forest, KNN: k-nearest neighbors, SVM: support vector machine, LR: logistic regression, AUC: area under the curve, SMOTE: synthetic minority oversampling technique, LAT: latency, MAX: maximum diameter, MAXCV: maximum constriction velocity, MDV: mean dilation velocity. * Unable to generate a best combination of PLR parameters due to class imbalance—models uniformly produced accuracy of 89%, sensitivity of 0%, specificity of 100%, AUC of 0.5, and F1 score of 0%.

**Table 5:** Best-performing PLR parameter combinations and models after SMOTE.

Model	Parameters	Accuracy	Sensitivity	Specificity	AUC	F1 Score
RF	LAT, %, MIN, MCV, MAXCV, MDV	91%	97%	86%	0.91	92%
KNN	LAT, MAX, MAXCV, MDV	89	92	86	0.89	89%
SVM	LAT, MAX, MAXCV	79	89	68	0.78	81%
LR	CHANGE, MCV, MDV	72%	78%	66%	0.72	74%

RF: Random forest, KNN: k-nearest neighbors, SVM: support vector machine, LR: logistic regression, AUC: area under the curve, LAT: latency, MAX: maximum diameter, MIN: minimum diameter, %: percent change in diameter, MCV: mean constriction velocity, MAXCV: maximum constriction velocity, MDV: mean dilation velocity.

## Data Availability

The data involved in this study may be viewed here: https://osf.io/wg9mu/?view_only=74600f47f1f546c8af7e5ffa14e4b5fb (accessed on 28 November 2024). The analysis code cannot be shared for intellectual property reasons.

## References

[1] Bryan M.A., Rowhani-Rahbar A., Comstock R.D., Rivara F., Seattle Sports Concussion Research C. (2016). Sports- and Recreation-Related Concussions in US Youth. Pediatrics.

[2] Stemper B.D., Harezlak J., Shah A.S., Rowson S., Mihalik J.P., Riggen L., Duma S., Pasquina P., Broglio S.P., McAllister T.W. (2022). Association between Preseason/Regular Season Head Impact Exposure and Concussion Incidence in NCAA Football. Med. Sci. Sports Exerc..

[3] Harmon K.G., Clugston J.R., Dec K., Hainline B., Herring S., Kane S.F., Kontos A.P., Leddy J.J., McCrea M., Poddar S.K. (2019). American Medical Society for Sports Medicine position statement on concussion in sport. Br. J. Sports Med..

[4] Harmon K.G., Whelan B.M., Aukerman D.F., Hwang C.E., Poddar S.K., DeLeo A., Elkington H.A., Garruppo G., Holliday M., Bruce J.M. (2024). Diagnosis of Sports-Related Concussion Using Symptom Report or Standardized Assessment of Concussion. JAMA Netw. Open.

[5] Kroshus E., Garnett B., Hawrilenko M., Baugh C.M., Calzo J.P. (2015). Concussion under-reporting and pressure from coaches, teammates, fans, and parents. Soc. Sci. Med..

[6] Echemendia R.J., Burma J.S., Bruce J.M., Davis G.A., Giza C.C., Guskiewicz K.M., Naidu D., Black A.M., Broglio S., Kemp S. (2023). Acute evaluation of sport-related concussion and implications for the Sport Concussion Assessment Tool (SCAT6) for adults, adolescents and children: A systematic review. Br. J. Sports Med..

[7] Bruce J.M., Thelen J., Meeuwisse W., Hutchison M.G., Rizos J., Comper P., Echemendia R.J. (2020). Use of the Sport Concussion Assessment Tool 5 (SCAT5) in professional hockey, part 2: Which components differentiate concussed and non-concussed players?. Br. J. Sports Med..

[8] Harmon K.G., Whelan B.M., Aukerman D.F., Bohr A.D., Nerrie J.M., Elkinton H.A., Holliday M., Poddar S.K., Chrisman S.P.D., McQueen M.B. (2022). Diagnostic accuracy and reliability of sideline concussion evaluation: A prospective, case-controlled study in college athletes comparing newer tools Ma and established tests. Br. J. Sports Med..

[9] King A., Devick S.T. (1976). The Proposed King-Devick Test and Its Relation to the Pierce Saccade Test and Reading Levels.

[10] King J., Friend C., Zhang D., Carr W. (2024). Comparative Performance of Three Eye-Tracking Devices in Detection of Mild Traumatic Brain Injury in Acute Versus Chronic Subject Populations. Mil. Med..

[11] Meier T.B., Huber D.L., Goeckner B.D., Gill J.M., Pasquina P., Broglio S.P., McAllister T.W., Harezlak J., McCrea M.A., for CARE Consortium Investigators (2024). Association of Blood Biomarkers of Inflammation with Acute Concussion in Collegiate Athletes and Military Service Academy Cadets. Neurology.

[12] Carrick F.R., Azzolino S.F., Hunfalvay M., Pagnacco G., Oggero E., D’Arcy R.C.N., Abdulrahman M., Sugaya K. (2021). The Pupillary Light Reflex as a Biomarker of Concussion. Life.

[13] Capo-Aponte J., Urosevich T.G., Walsh D.V., Temme L.A., Tarbett A.K. (2013). Pupillary Light Reflex as an Objective Biomarker for Early Identification of Blast-Induced mTBI. J. Spine.

[14] Thiagarajan P., Ciuffreda K.J. (2015). Pupillary responses to light in chronic non-blast-induced mTBI. Brain Inj.

[15] Truong J.Q., Ciuffreda K.J. (2016). Comparison of pupillary dynamics to light in the mild traumatic brain injury (mTBI) and normal populations. Brain Inj.

[16] Capo-Aponte J.E., Beltran T.A., Walsh D.V., Cole W.R., Dumayas J.Y. (2018). Validation of Visual Objective Biomarkers for Acute Concussion. Mil. Med..

[17] Hsu J., Stec M., Ranaivo H.R., Srdanovic N., Kurup S.P. (2021). Concussion Alters Dynamic Pupillary Light Responses in Children. J. Child. Neurol..

[18] Master C.L., Podolak O.E., Ciuffreda K.J., Metzger K.B., Joshi N.R., McDonald C.C., Margulies S.S., Grady M.F., Arbogast K.B. (2020). Utility of Pupillary Light Reflex Metrics as a Physiologic Biomarker for Adolescent Sport-Related Concussion. JAMA Ophthalmol..

[19] Bower M.M., Sweidan A.J., Xu J.C., Stern-Neze S., Yu W., Groysman L.I. (2021). Quantitative Pupillometry in the Intensive Care Unit. J. Intensive Care Med..

[20] Signoretti S., Lazzarino G., Tavazzi B., Vagnozzi R. (2011). The pathophysiology of concussion. PM R.

[21] McAnany J.J., Smith B.M., Garland A., Kagen S.L. (2018). iPhone-based Pupillometry: A Novel Approach for Assessing the Pupillary Light Reflex. Optom. Vis. Sci..

[22] Piaggio D., Namm G., Melillo P., Simonelli F., Iadanza E., Pecchia L. (2021). Pupillometry via smartphone for low-resource settings. Biocybern. Biomed. Eng..

[23] McGrath L.B., Eaton J., Abecassis I.J., Maxin A., Kelly C., Chesnut R.M., Levitt M.R. (2022). Mobile smartphone-based digital pupillometry curves in the diagnosis of traumatic brain injury. Front. Neurosci..

[24] Maxin A.J., Lim D.H., Kush S., Carpenter J., Shaibani R., Gulek B.G., Harmon K.G., Mariakakis A., McGrath L.B., Levitt M.R. (2024). Smartphone Pupillometry and Machine Learning for Detection of Acute Mild Traumatic Brain Injury: Cohort Study. JMIR Neurotech.

[25] Memmini A.K., Mosesso K.M., Perkins S.M., Brett B.L., Pasquina P.F., McAllister T.W., McCrea M.A., Broglio S.P., Investigators C.C. (2023). Premorbid Risk Factors and Acute Injury Characteristics of Sport-Related Concussion Across the National Collegiate Athletic Association: Findings from the Concussion Assessment, Research, and Education (CARE) Consortium. Sports Med..

[26] Patricios J.S., Schneider K.J., Dvorak J., Ahmed O.H., Blauwet C., Cantu R.C., Davis G.A., Echemendia R.J., Makdissi M., McNamee M. (2023). Consensus statement on concussion in sport: The 6th International Conference on Concussion in Sport-Amsterdam, October 2022. Br. J. Sports Med..

[27] Maxin A.J., Gulek B.G., Lee C., Lim D., Mariakakis A., Levitt M.R., McGrath L.B. (2023). Validation of a Smartphone Pupillometry Application in Diagnosing Severe Traumatic Brain Injury. J. Neurotrauma.

[28] Maxin A.J., Kush S., Gulek B.G., Winston G.M., Chae J., Shaibani R., McGrath L.B., Abecassis I.J., Levitt M.R. (2024). Smartphone pupillometry for detection of cerebral vasospasm after aneurysmal subarachnoid hemorrhage. J. Stroke Cerebrovasc. Dis..

[29] Maxin A.J., Gulek B.G., Chae J., Winston G., Weisbeek P., McGrath L.B., Levitt M.R. (2023). A smartphone pupillometry tool for detection of acute large vessel occlusion. J. Stroke Cerebrovasc. Dis..

[30] Mariakakis A., Baudin J., Whitmire E., Mehta V., Banks M.A., Law A., McGrath L., Patel S.N. (2017). PupilScreen: Using smartphones to assess traumatic brain injury. Proc. ACM Interact. Mob. Wearable Ubiquitous Technol..

[31] Rashidi H.H., Tran N.K., Betts E.V., Howell L.P., Green R. (2019). Artificial Intelligence and Machine Learning in Pathology: The Present Landscape of Supervised Methods. Acad. Pathol..

[32] Chawla N.V., Bowyer K.W., Hall L.O., Kegelmeyer W.P. (2002). SMOTE: Synthetic minority over-sampling technique. J. Artif. Intell. Res..

[33] Garcia G.P., Broglio S.P., Lavieri M.S., McCrea M., McAllister T., Investigators C.C. (2018). Quantifying the Value of Multidimensional Assessment Models for Acute Concussion: An Analysis of Data from the NCAA-DoD Care Consortium. Sports Med..

[34] Resch J.E., Brown C.N., Schmidt J., Macciocchi S.N., Blueitt D., Cullum C.M., Ferrara M.S. (2016). The sensitivity and specificity of clinical measures of sport concussion: Three tests are better than one. BMJ Open Sport Exerc. Med..

[35] Patricios J., Schneider G.M., van Ierssel J., Purcell L.K., Davis G.A., Echemendia R.J., Fremont P., Fuller G.W., Herring S., Harmon K.G. (2023). Sport Concussion Office Assessment Tool—6. Br. J. Sports Med..

[36] Naidu D., Borza C., Kobitowich T., Mrazik M. (2018). Sideline Concussion Assessment: The King-Devick Test in Canadian Professional Football. J. Neurotrauma.

[37] Fuller G.W., Cross M.J., Stokes K.A., Kemp S.P.T. (2019). King-Devick concussion test performs poorly as a screening tool in elite rugby union players: A prospective cohort study of two screening tests versus a clinical reference standard. Br. J. Sports Med..

[38] Heyming T., Knudsen-Robbins C., Schomberg J., Hayakawa J., Lara B., Bacon K., Valdez B., Wickens M., Shelton S.K., Romain J. (2024). Evaluation of Quantitative Pupillometry in Acute Postinjury Pediatric Concussion. Pediatr. Neurol..

[39] Oeur A., Mull M., Riccobono G., Arbogast K.B., Ciuffreda K.J., Joshi N., Fedonni D., Master C.L., Margulies S.S. (2023). Pupillary Light Response Deficits in 4-Week-Old Piglets and Adolescent Children after Low-Velocity Head Rotations and Sports-Related Concussions. Biomedicines.

[40] Lee M.H., Mitra B., Pui J.K., Fitzgerald M. (2018). The use and uptake of pupillometers in the Intensive Care Unit. Aust. Crit. Care.

[41] Gramkow M.H., Clemmensen F.K., Sjaelland N.S., Waldemar G., Hasselbalch S.G., Frederiksen K.S. (2024). Diagnostic performance of light reflex pupillometry in Alzheimer’s disease. Alzheimers. Dement..

[42] Kaifie A., Reugels M., Kraus T., Kursawe M. (2021). The pupillary light reflex (PLR) as a marker for the ability to work or drive—A feasibility study. J. Occup. Med. Toxicol..

[43] Huyghe T., Calleja-Gonzalez J., Bird S.P., Alcaraz P.E. (2024). Pupillometry as a new window to player fatigue? A glimpse inside the eyes of a Euro Cup Women’s Basketball team. Biol. Sport.

